# Digenean larvae—the cause and beneficiaries of the changes in host snails’ thermal behavior

**DOI:** 10.1007/s00436-014-4276-z

**Published:** 2015-01-07

**Authors:** Elżbieta Żbikowska, Janusz Żbikowski

**Affiliations:** 1 Department of Invertebrate Zoology, Faculty of Biology and Environment Protection, Nicolaus Copernicus University, Toruń, Poland; 2 Department of Hydrobiology Faculty of Biology and Environment Protection, Nicolaus Copernicus University, Toruń, Poland

**Keywords:** Digenean larvae, Thermal behavior, Snail host, Notocotylids, Echinostomes

## Abstract

Parasite-induced changes in host’s thermal preferences not only can be interpreted as a physiological defense response of the host but also can represent a pathological manifestation of the parasite. Both may become established in host-parasite relationships if they are beneficial for at least one of the counterparts. This study investigates parasite-induced changes in the thermoregulatory behavior of first intermediate hosts of Digenea (i.e. *Lymnaea stagnalis* and *Planorbarius corneus*), infected with Notocotylidae or Echinostomatidae larvae. The investigated parasite species developed different transmission strategies outside the body of a snail, which may imply a different effect on the behavior of their hosts. *Notocotylus attenuatus* in *L. stagnalis* and *Notocotylus ephemera* in *P. corneus* produce symptoms of anapyrexia, prolonging the lifespan of their hosts. By contrast, *Echinoparyphium aconiatum* in *L. stagnalis* and *Echinostoma spiniferum* in *P. corneus* interfere with defensive thermoregulatory behavior of host snails, causing their accelerated death. The results of laboratory research indicate that thermal preferences of the snails infected with all investigated trematodes facilitate the transmission of the parasites in environment.

## Introduction

The relationship between snails and larval digenetic trematodes constitutes a model to study host-parasite interactions. Nearly 90 % of 25,000 described species of trematodes use snails as first obligate hosts. The relationship has formed over the last 200 million years (Blair et al. 2001). By some researchers, the behavioral aspect of the mutual adaptation in the snail-trematode relationship is defined as the ability of the parasite to manipulate the host. Numerous examples of this “manipulation” were presented by Moore (2002). After that, a comprehensive analysis of the available data led Thomas et al. (2005) to the conclusion that the manipulation is a fact which, however, requires a thorough explanation. Specialists in ecological parasitology, ecophysiology, and immunology have been engaged in investigating the issue (Esch et al. 2002; Levri et al. 2007; Walker 2006).

Altered behavior of snails caused by the presence of larval digenetic trematodes was interpreted as an opportunity to increase the propagation of the parasites. Levri (1999) noticed that a *Microphallus* sp. determined the behavior of *Potamopyrgus antipodarum* towards predators which were the potential hosts of the parasites. Similarly, Bernot (2003) observed that *Physa integra* infected with larval Paramphistomidae or Cathaemasidae exposed themselves to predators which were to become hosts of the parasites in the next stage of their lifecycle. Miura et al. (2006) noticed that parasitic larvae contributed to the creation of new ecological niches and a better use of food consumed by host snails. Finally, Sandland and Minchella (2003) explained the reduced mobility of snails infected with trematodes as a way of allocating host energy to the production of parasitic larvae.

A very interesting behavioral aspect of the research on the defense responses of snails focused mainly on the fact that these ectotherms displayed a number of adaptations protecting them from death from overheating or freezing (Nowakowska 2011; Ademolu et al. 2011; Marshall et al. 2011). The analysis of snails’ behavior in both thermally variable natural environments and the laboratory (Achaval et al. 2005) indicates that snails are capable of thermoreception and their thermoregulatory responses are not random. Research on thermal preferences of different snail species using a thermal gradient indicates that in the laboratory, snails tend to select temperatures much higher than those prevailing in their natural environment (Chernin 1967; Ross and Ultsch 1980; Żbikowska and Cichy 2012). This surprising fact can be explained by evolutionary heritage: adaptations to warm water conditions developed by the ancestors of modern snail species were passed in the genes. They are responsible for the fact that a small temperature drop inhibits vital functions to a greater extent than a temperature increase (both compared to the optimum) (Parashar and Rao 1991; Matsukura et al. 2009).

Temperature determines the physiology of both counterparts in a snail-trematode relationship. To assess which counterpart benefits more from the surrounding temperature, one needs to investigate a variety of host-parasite relationships, particularly in freshwater ecosystems. The interactions between freshwater snails and trematodes provide diverse material on reproductive strategies of parasites. Due to annual temperature variations, freshwater ecosystems located in the temperate zone constitute a testing ground for studying thermoregulatory responses of ectotherms. Similarly to their parasistic trematodes, freshwater snails inhabiting this area have developed a number of adaptations determining their survival in a thermally variable environment.

For thermobehavioral study, we chose two pulmonate snail species: *Planorbarius corneus* and *Lymnaea stagnalis*—common in freshwater ecosystems. In the temperate zone, both species serve as first intermediate hosts for several species of Digenea (Żbikowska and Nowak 2009; Cichy et al. 2011). A majority of these trematodes, including *Notocotylus attenuatus* and *Echinoparyphium aconiatum* (specific for *L. stagnalis*) and *Notocotylus ephemera* and *Echinostoma spiniferum* (specific for *P. corneus*), are castrating parasites (Żbikowska 2011). Released from the first intermediate hosts, cercariae of both studied echinostomes may select snail species, including *L. stagnalis* or *P. corneus*, also as their second intermediate hosts (Faltýnková et al. 2007, 2008). By contrast, cercaria of notocotylids released from snails into water cast off their tails, encyst, and develop into metacercariae out of a host body. On some occasions, the cercariae encyst and develop into metacercariae within the first intermediate hosts (Żbikowska 2007). Description of life cycles of parasites used in the study was presented by Yamaguti (1938), Zdarska (1964) and Nasincova (1992). Having acknowledged a strong relationship between larval trematodes and their first intermediate hosts, we suggest that thermoregulatory behavior of host snails is related to the reproductive strategy of Digenea which develop inside their bodies.

## Materials and methods

### Parasitic diagnosis and preparing snails for an experiment in a thermal gradient

Sexually mature *L. stagnalis* and *P. corneus* were collected from May to October during three growing seasons from eutrophic lakes located in the Iława Lake Region, incl. Lake Jeziorak (N53°37′ E19°33′) and Lake Tynwałdzkie (N53° 66′ E19° 63′), Brodnica Lake Region, incl. Lake Strażym (N53° 20′ E19°26′), Lake Bachotek (N53° 28′ E19° 47′), and Lake Zbiczno (N53° 20′ E19°24′), and in the Tuchola Forest from Lake Charzykowskie (N53° 46′ E17°30′). During the research period, we collected 3105 specimens of *L. stagnalis* and 2514 specimens of *P. corneus*. In order to verify trematode invasion in laboratory, snails were placed individually in small beakers filled with tap water for 1–2 h. The species of the released cercariae (i.e., during patent period of invasion) were determined according to Nasincova (1992) and Niewiadomska et al. (1997). Seventy-four snails were found to be naturally infected with *N. attenuatus*, 69 snails with *N. ephemera*, 81 snails with *E. aconiatum*, and 91 snails with *E. spiniferum*. Snails releasing cercariae of *N. attenuatus* and *N. ephemera* were collected mainly in spring and fall, whereas most patent invasions of *E. aconiatum* and *E. spiniferum* were recorded in summer (Table 1).

**Table 1:** Presence of all investigated species of trematodes in the investigated populations of *L. stagnalis* and *P. corneus* throughout the entire research season

Lakes	Number of *L. stagnalis* individuals				Number of *P. corneus* individuals			
	Σ	Infected	With *N. attenuatus*	With *E. aconiatum*	Σ	Infected	With *N. ephemera*	With *E. spiniferum*
Jeziorak	621	368	18	21	583	239	11	19
Tynwałdzkie	653	499	26	12	661	521	21	20
Strażym	327	152	11	23	321	96	10	11
Bachotek	699	238	6	6	281	111	9	10
Zbiczno	361	159	4	11	367	125	7	15
Charzykowskie	444	267	9	8	301	164	11	16
TOTAL	3105	1683	74	81	2514	1256	69	91

Specimens infected with *N. attenuatus*, *N. ephemera*, *E. aconiatum*, or *E. spiniferum* were separated and acclimated for 1 week at 19 °C (Żbikowska and Cichy 2012), abundantly fed lettuce. The experiments involved nine groups of snails: four groups of snails infected with trematodes and shedding cercariae (patent invasion), four groups of snails infected with trematodes but not shedding cercariae (pre-patent invasion), and the control group consisting of uninfected snails. The absence of parasites or stage of invasion was confirmed by dissecting the snails after the experiment.

### Experiment 1. Observations in a thermal gradient

**Fig. 1:**
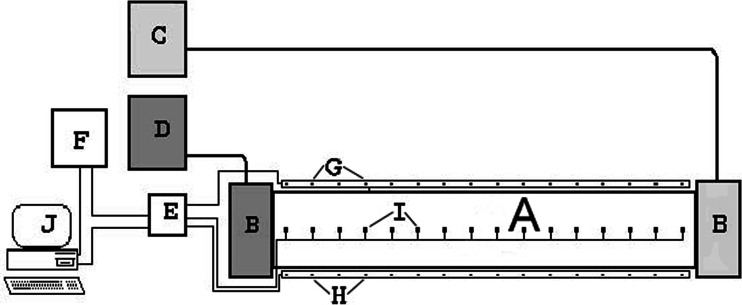
Experimental setup for recording thermal behavior of snails (according to Grodzicki and Caputa 2005): *A* thermal gradient chamber (water level 0.5 cm), *B* fluid chambers, *C* thermostat, *D* cryostat, *E* electronic switch of thermocouples, *F* scanner, *G* transmitters of infrared radiation, *H* receivers of infrared radiation, *I* thermocouples, and *J* computer

The total number of snails, whose behavior in a thermal gradient was recorded automatically, included two control groups of uninfected snails (60 individuals of *P. corneus* and *L. stagnalis*), and 156 snails infected with larval trematodes: of *N. attenuatus*, *E. aconiatum*, *N. ephemera*, or *E. spiniferum*. A majority of the investigated snails shed cercariae prior to the experiment in the gradient. Pre-patent invasion was confirmed in 18 *L. stagnalis* and in 15 *P. corneus*. The snails were placed individually in a rectangular oblong thermal gradient (temperature range +4 to +38 °C) (Fig. 1). The temperature gradient was generated by circulating fluids (Petrygo Q and water, respectively) controlled by PolyScience ultrathermostats. The experiment was carried out in a room with the temperature of 19–20 °C. Each snail was used only once, and the number of repeats is shown in Table 2.

**Table 2:** Thermal preferences of the investigated *L. stagnalis* and *P. corneus*

Snails	Invasion	Parasite	Number	Avg. 24th temperature [°C]*	SE
*Lymnaea stagnalis*	No (control)	0	30	25.0a	0.3
	Pre-patent	*N. attenuatus*	8	26.1ac	0.5
		*E. aconiatum*	10	24.7ac	0.4
	Patent	*N. attenuatus*	31	17.6b	0.3
		*E. aconiatum*	31	24.9ac	0.3
*Planorbarius corneus*	No (control)	0	30	25.6a	0.7
	Pre-patent	*N. ephemera*	8	25.2ac	0.4
		*E. spiniferum*	7	24.8ac	0.3
	Patent	*N. ephemera*	31	17.3b	0.3
		*E. spiniferum*	30	26.2ac	0.2

*P < 0.001, different letters near avg. 24th (average daily) temperature label difference statistical significant; Tukey test with different *N*

The results were automatically recorded and computed at 3-min intervals during 24 h using a custom data acquisition computer program GRAD. For the data analysis, excel plotting, and presentation, the temperature recordings of the control and experimental snails’ groups were pooled into 24-h averages (avg. 24th).

### Experiment 2. Survival rate of snails kept at a constant temperature

After the experiment in a thermal gradient, nine groups of snails including the control groups (40 *L. stagnalis* and 40 *P. corneus*) and the snails infected with *N. attenuatus* (24 *L. stagnalis*), *N. ephemera* (24 *P. corneus*), *E. aconiatum* (24 *L. stagnalis*), or *E. spiniferum* (24 *P. corneus*) were kept at a constant temperature. Half of the snails of each group were kept at 18 °C and the other half at 26 °C (values near average temperatures from selected by snails during experiment 1). The number of cercariae released from infected snails into the water was counted daily. The snails were transferred to new beakers, and the cercariae were counted after being fixed with ethanol.

### Experiment 3. Survival rate of cercariae released by host snails

Freshly emerged cercariae were placed in Petri dishes (50 larvae in a dish) and left in incubators at 18 or 26 °C. The time needed for the transformation of *N. attenuatus* and *N. ephemera* into metacercariae was monitored regularly. Every hour, motionless cercariae of *E. aconiatum* and *E. spiniferum* were counted and removed from the dishes. The number of the remaining live larvae was then calculated. All observations were performed in five replicates.

### Experiment 4. Invasiveness of echinocercariae

To study the echinostome larvae invasiveness, *P. antipodarum* individuals were used, due to their susceptibility to invasion with these parasites (Żbikowski and Żbikowska 2009). Freshly emerged cercariae of *E. aconiatum* or *E. spiniferum* were placed in beakers together with *P. antipodarum* snails (30 cercariae and 10 *P. antipodarum* in a beaker) (3.0 ± 0.2 mm). Half of the beakers were incubated at 18 °C and the other half at 26 °C. Cercariae floating in the beakers were counted after 20, 40, 60, 120, and 140 min. The final stage of the experiment involved dissecting the snails in order to count metacercariae in their tissues.

All observations were performed in five replicates.

### Statistical analysis

The average daily temperatures selected by the investigated groups of snails were calculated, and the results were analyzed by two-way ANOVA (factor 1: snail species: *L. stagnalis* or *P. corneus*; factor 2: type of invasion none, patent, or pre-patent invasion of notocotylidae, patent or pre-patent invasion of echinostomatidae) followed by Tukey multiple comparisons of means if significant.

Three-way ANOVA was applied to analyze the survival rate of snails and cercariae shedding (factors: snail species, type of parasitic invasion, temperature). It examined the lifespan of snails and the number of cercariae released by the infected specimens. To determine the significance of differences *T* tests were performed followed by Bonferroni test.

To assess the survival rate of echinocercariae at two temperatures, LT_50%_ value was calculated using a probit regression. The time needed by the parasites to penetrate *P. antipodarum* and the number of metacercariae were analyzed using the *T* test.

## Results

The experiment in thermal gradient revealed differences between the behavior of the infected and the uninfected specimens. The snails in the control group (uninfected) actively penetrated the thermal gradient occupying its warmer end for longer periods of time. The snails shedding cercariae of *N. attenuatus* or *N. ephemera* selected the cooler end of the gradient. The snails infected with *E. aconiatum* or *E. spiniferum* and the snails with the pre-patent invasion of *N. attenuatus* and *N. ephemera* were less active and, once they moved to the warmer end of the gradient, remained there until the end of the experiment. The analysis of the average daily temperatures indicates that snails’ thermal preferences were not determined by the species itself (*P* = 0.58) but by the parasitic invasion *F*_4, 206_ = 194.9 (*P* < 0.001). The average daily temperatures calculated for each group of snails are presented in Table 2.

The research on the survival rate of snails kept at a constant temperature of 18 or 26 °C indicates that both the temperature and the parasitic invasion affected the lifespan of the animals (*F*_2, 164_ = 72.1; *P* < 0.001). The majority of the investigated snails lived longer at a lower temperature. The presence of parasites usually decreased the lifespan of the hosts. Uninfected *P. corneus* kept at 18 °C had the longest lifespan while *P. corneus* infected with *E. spiniferum* kept at 26 °C had the shortest. Larval notocotylids affected the hosts’ lifespan to a lesser extent than echinostomes. Moreover, the impact of echinostomes on the lifespan of the snails was not related to the temperature in the gradient. The results are presented in Table 3.

**Table 3:** The average lifespan of snails used in the experiment, kept at constant temperatures

Snail	Invasion	Number	Temperature [°C]	Average lifespan [days]*	SE
*L. stagnalis*	No invasion (control)	20	18	69a	5.0
		20	26	21b	1.0
	*N. attenuatus*	12	18	52c	2.0
		12	26	19b	1.0
	*E. aconiatum*	12	18	10b	0.8
		12	26	9b	0.8
*P. corneus*	No invasion (control)	12	18	267d	5.6
		12	26	89e	3.1
	*N. ephemera*	12	18	173f	4.5
		12	26	18b	1.0
	*E. spiniferum*	12	18	14bg	1.1
		12	26	6g	0.4

*P < 0.01, values statistically different are labeled with different letters (a–g); LSD Fisher post hoc test

The number of cercariae released by the snails kept at a constant temperature depended on the temperature, the host species, and the parasite species. Due to the fact that statistical analysis showed the significance of the interaction between the snail species and the type of parasite invasion (*P* < 0.001), *T* test was performed for respective pairs of values. The results indicate that echinocercariae released from the infected snails were more abundant than notocotylid larvae. Moreover, a higher number of notocotylid cercariae were released at a lower temperature (18 °C). *E. aconiatum* echinocercariae (parasitizing *L. stagnalis*) were more abundant than *E. spiniferum* (parasitizing *P. corneus*). More notocotylid cercariae were released from *P. corneus* (*N. ephemera*) than from *L. stagnalis* (*N. attenuatus*). Average numbers of released cercariae are presented in Table 4.

**Table 4:** The number of cercariae released by host snails which were kept at a constant temperature

Snail	Invasion	Temp. [°C]	Average number of cercariae ± SD [larvae/snail]	Statistical significant differences between compared groups
*L. stagnalis*	*N. attenuatus*	18	112 ± 40	*LN*18/*LN*26***
		26	63 ± 50	*LN*18/*LE*18***
	*E. aconiatum*	18	1101 ± 34	*LN*26/*LE*26***
		26	998 ± 12	*LN*18/*PN*18***
*P. corneus*	*N. ephemera*	18	248 ± 60	*LE*18/*PE*18***
		26	171 ± 50	*LE*26/*PE*26***
	*E. spiniferum*	18	504 ± 15	*PN*18/*PN*26***
		26	421 ± 13	*PN*18/*PE*18***

*LN18 L. stagnalis* with *N. attenuatus* at 18 °C, *LN26 L. stagnalis* with *N. attenuatus* at 26 °C, *LE18 L. stagnalis* with *E. aconiatum* at 18 °C, *LE26 L. stagnalis* with *E. aconiatum* at 26 °C, *PN18 P. corneus* with *N. ephemera* at 18 °C, *PN26 P. corneus* with *N. ephemera* at 26 °C, *PE18 P. corneus* with *E. spiniferum* at 18 °C, *PE26 P. corneus* with *E. spiniferum* at 26 °C

***P < 0.0001, data analyzed using *T* test followed by Bonferroni test

The results of the probit regression indicate that temperature significantly affected the survival rate of cercariae released by host snails. At 18 °C, LT_50%_ was 13.7 h for *E. aconiatum* and 13.8 h for *E. spiniferum*. At 26 °C, it was 5.2 and 5.5 h, respectively. However, it did not affect the transformation of notocotlid cercariae into metacercariae, fully shaped 30 min after leaving the body of snails.

Temperature determined the amount of time needed by echinocercariae to invade second intermediate hosts. After 140 min, all freshly released echinocercariae penetrated *P. antipodarum*, placed in the same beakers filled with water. The results presented in Table 5 indicate that cercariae of both echinostomes needed less time to invade second intermediate host snails when the temperature was higher. The dissection of snails showed no statistically significant differences between the numbers of metacercariae formed within their bodies (Table 5).

**Table 5:** Values illustrating behavior of echinocercariae towards *P. antipodarum* at two temperatures

Duration of the experiment [min]	Number of cercariae *E. aconiatum* in water			Number of cercariae *E. spiniferum* in water		
	Mean ± SD			Mean ± SD		
	Temp. 18 °C	Temp. 26 °C	*P* ^a^	Temp. 18 °C	Temp. 26 °C	*P* ^a^
0	30	30		30	30	
20	26.0 ± 1.30	14.4 ± 2.40	0.003	24.8 ± 2.40	10.6 ± 2.20	0.000
40	16.4 ± 1.50	7.8 ± 1.85	0.007	14.2 ± 1.24	2.2 ± 1.32	0.000
60	5.8 ± 1.43	2.6 ± 0.81	0.087	6.2 ± 0.97	0.4 ± 0.40	0.001
80	3.6 ± 1.08	0.6 ± 0.40	0.031	3.2 ± 0.97	0	0.012
100	1.8 ± 0.58	0.2 ± 0.20	0.032	1.8 ± 0.97	0	0.098
120	0.8 ± 0.58	0	0.207	1.4 ± 0.75	0	0.233
140	0	0		0	0	
Number of metacercariae mean ± SD	21 ± 1.9	18 ± 2.3	>0.05	22 ± 1.3	20 ± 1.3	>0.05

^a^ *P* and *T test*; significance of differences between the values calculated for two temperatures

## Discussion

The issue of how parasite-induced behavioral alterations of host snails can benefit trematode parasites has been thoroughly analyzed (Curtis 1987, 2007; Huxham et al. 1995; Levri and Lively 1996; Levri and Fisher 2000; Davies and Knowles 2001; Bernot 2003; Kamiya and Poulin 2012). Most examples provided by the authors illustrate the failure of the self-preservation instinct in the infected hosts—donors exposing themselves to predatory hosts—recipients of the parasites. Only several studies investigating “parasitic manipulation” include experimental data which indicate a physiological mechanism of these deviations (Levri and Lively 1996; Levri and Fisher 2000).

Our observations indicate that all snails with pre-patent parasite invasion moved to the warmer end of the gradient. There is no doubt that increased temperature promotes the development of trematodes, as has been pointed out by other authors (Liao 1993; Fried 1997; Paull and Johnson 2011). The average daily temperature selected by the snails infected with redia and/or sporocysts of *N. attenuatus* or *E. spiniferum* was even slightly higher than the average daily temperature calculated for the control snails. However, the differences were not statistically significant and cannot be regarded as the symptoms of behavioral fever.

The absence of thermoregulatory response of the investigated snails with pre-patent trematode invasion did not result from the inability of the invertebrate ectotherms to produce it. The symptoms of behavioral fever have been identified not only in arthropods (see the review: de Roode and Lefebvre 2012) but also in molluscs (Żbikowska et al. 2013a, b). We recorded them in wintering *P. corneus* inoculated with natural or synthetic pyrogens (zymosan, LPS, poly I:C). The inability to produce fever symptoms by snails with prepatent trematode invasion may have been caused by the inhibitory effect of redia and/or sporocysts. Parasite-induced inhibition of behavioral fever in invertebrates generally capable of thermoregulation was described by Ballabeni et al. (1995). They observed that *Drosophila* sp. did not respond with behavioral fever to the nematode invasion, although culturing the infected insects at raised temperatures had a therapeutic effect. These example as well as numerous publications indicate that the interaction of a host-parasite relies on a compromise between host’s fitness and parasite’s needs (André et al. 2003; Salvaudon et al. 2005; McQuaid and Britton 2013).

Very different results were obtained for some snails with patent parasite invasion. The analysis of thermoregulatory responses of *P. corneus* and *L. stagnalis* shedding notocyclid cercariae shows that both snail species respond with symptoms of behavioral anapyrexia (Table 2). Similar results were described also in other snail-trematode associations (Żbikowska and Cichy 2012). This preference for lower temperatures demonstrated by snails in critical moments of trematode invasion was also observed by Lefcort and Bayne (1991), who, however, did not interpret this reaction as a defense mechanism. The results of our observations reveal that a decreased temperature did not cure the snails but prolonged their lifespan more significantly than a raised temperature (Table 3). This may suggest that snails were the beneficiaries of these thermoregulatory responses, but it should be mentioned that parasitic castration prevented them from increasing their population. On the other hand, in the experiment, the hosts of *N. attenuatus* or *N. ephemera* kept at a decreased temperature released significantly more cercariae (which immediately transform into metacercariae outside host body) compared to the hosts kept at an elevated temperature (Table 4). The point of view that the extended lifespan of the infected hosts turned out to be beneficial mainly for the trematodes was presented also by Graczyk and Shiff (1994).

On the other hand, we noticed no symptoms of anapyrexia in the snails releasing echinocercariae (Table 2). The hosts of echinostomes selected temperatures within the range favored by control snails regardless of the stage of the parasite development. A different effect of notocotylids and echinostomes on the snails of the same species with the patent parasite invasion may result from the differences in the transmission strategies of the parasites. Echinostome species are known to exploit their first hosts intensively but for a short period of time. Field surveys (Curtis 2003; Frendensborg et al. 2005) indicate that a long-term exposure to elevated temperatures is poorly tolerated by echinostome infected hosts. Numerous species of snails may become second intermediate hosts of echinostomes—including those that play a role of first intermediate hosts. Our earlier observations (Żbikowska 2006) suggest that second intermediate hosts of echinostomes are recruited mostly from snails free from the invasion by rediae and/or sporocysts. The penetration of hosts with patent trematode invasions may prove unsuccessful due to the high mortality of the attacked snails (Galaktionov and Dobrovolskiy 2003). The experiments using a thermal gradient demonstrates that the uninfected specimens select microenvironments with an increased temperature, usually higher than that prevailing in their natural environment (Ross and Ultsch 1980; Żbikowska 2006, 2011; Żbikowska and Cichy 2012). It can therefore be speculated that similar thermal preferences of both uninfected and echinocercariae shedding groups of snails facilitate the transmission of larvae into second intermediate hosts. It is worth underlining that although both *E. aconiatum* in *L. stagnalis* and *E. spiniferum* in *P. corneus* inhibited behavioral anapyrexia in snails with patent invasion, which caused the hosts’ death in a relatively short time (Table 3), this did not reduce the number of released cercariae (Table 4). Moreover, the research on the invasion mechanisms of both echinostomes suggests that their cercariae, kept at an increased temperature, tended to find and penetrate the second host more easily, thus significantly shortening the time they spent in the external environment (Table 5). Our experiment shows that the inhibition of behavioral anapyrexia in echinostome-infected snails benefits parasites.

The research indicates that the snails’ defense responses are controlled by the parasites invading their bodies and the understanding the mechanism of this process needs further study. Our observation indicates that capable of defensive thermoregulatory behavior, the hosts adapt only those defensive strategies which favor the propagation of trematodes.
